# Promoting Social Connectedness Through Interbrain Neurofeedback

**DOI:** 10.1111/nyas.70135

**Published:** 2025-11-10

**Authors:** Xiaojun Cheng, Rongbin Zhang, Phoebe Chen, Ziyuan Song, Feng Cheng, Suzanne Dikker, Yafeng Pan

**Affiliations:** ^1^ School of Psychology Shenzhen University Shenzhen China; ^2^ Department of Psychology New York University New York New York USA; ^3^ Department of Experimental Psychology Ghent University Ghent Belgium; ^4^ Department of Psychology and Behavioral Sciences Zhejiang University Hangzhou China; ^5^ The State Key Lab of Brain‐Machine Intelligence Zhejiang University Hangzhou China

**Keywords:** EEG hyperscanning, interbrain coupling, interbrain neurofeedback, shared mental process, social connectedness

## Abstract

Humans are inherently driven to form meaningful relationships, yet attempts at social connection often fall short or fail. This study investigates whether social connectedness can be improved by modulating interbrain coupling—a neural correlate of successful social interactions—through neurofeedback. Using a multibrain computer interface that visualized, in real time, the degree to which dyad members' electroencephalography (EEG) signals synchronized, dyads were randomly assigned to receive either neurofeedback or sham feedback generated from random signals. Compared with the sham group, dyads receiving neurofeedback showed greater interbrain coupling, and increases in coupling were associated with stronger feelings of social connectedness. A chain‐mediation analysis suggested that the experience of enhanced social connectedness was driven by a sense of joint control and shared intentionality. Together, these findings demonstrate the potential of interbrain neurofeedback to modulate interbrain coupling and support key components of social connectedness.

## Graphical abstract

This study shows that real‐time dyadic neurofeedback can enhance interbrain coupling and, importantly, increase social connectedness between individuals. Participants who viewed visualizations of their brainwave synchrony reported stronger feelings of connectedness, possibly driven by a sense of joint control and shared intentionality.

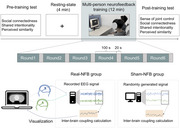

## 1 Introduction

Social connectedness is essential for our psychological and physical well‐being: it provides us with a sense of identity, purpose, and belonging [1, 2], and the lack of rewarding social interactions has been linked to adverse health effects [3, 4]. Individuals who perceive a strong sense of social connectedness in general often experience deep emotional bonds with others, readily empathize with them, view them as approachable and friendly, and actively engage in social groups and activities [5, 6]. A growing body of hyperscanning studies suggests that interbrain coupling—the alignment of neural activity between interacting individuals—may support core aspects of social connectedness. Interbrain coupling has been observed in diverse interactive contexts, including interpersonal communication [7, 8], interactive learning [9, 10], and economic decision making [11, 12]. Notably, interbrain coupling during interactions has been linked to downstream prosocial outcomes such as bonding, helping, and cooperation [11, 13], suggesting that moment‐to‐moment neural alignment may reflect or even facilitate the experience of social connectedness. However, some of these associations may reflect pre‐existing social ties or task‐driven similarities, rather than active alignment.

Beyond correlational findings, recent work has examined whether interbrain coupling can be externally modulated, raising the possibility of deliberately enhancing neural synchrony to influence social outcomes. For example, experimentally induced interbrain coupling has been linked to increased empathy [14], coordination [15], and learning success [16]. These findings suggest that modulation of interbrain coupling may lead to cognitive and affective states relevant to social functioning, in accordance with the shared representation and predictive coding theories in social contexts [17, 18, 19]. Building on these findings, an important next step is to examine whether externally enhancing interbrain coupling can foster social connectedness, and to understand the underlying mechanisms of this effect.

One potential mechanism involves the alignment of shared mental processes. Shared mental processes refer to the alignment of cognitive and emotional processes between individuals encompassing various psychological activities associated with “theory of mind,” and include at least two theoretically distinct but complementary pathways: shared intentionality [20] and perceived similarity  [21]. Shared intentionality involves a mutual recognition of goals and actions within a dyad or a group [22], whereas perceived similarity reflects the extent to which individuals regard one another as psychologically alike—sharing similar perspectives, values, or affective states [23, 24].

Both processes are closely related to social bonding [20, 25], though they may operate through different social cues and contexts. In particular, while shared intentionality may arise from active goal coordination, perceived similarity often emerges naturally in shared environments or during parallel tasks, even without explicit coordination. For example, individuals engaged in the same activity or coattending a stimulus may develop a stronger sense of similarity, which also promotes social bonding. Prior studies provide partial support for this framework. Some studies associate task‐induced interbrain coupling primarily with shared intentionality [20], while others highlight neural alignment linked to perceived similarity in naturalistic social contexts  [21]. Given this, we propose that interbrain coupling may enhance social connectedness via both pathways. To test this, our study includes shared intentionality and perceived similarity as complementary mediators in a controlled multibrain neurofeedback paradigm.

In recent years, neurofeedback has emerged as a promising intervention for regulating brain activity in individuals [26, 27]. During neurofeedback interventions, participants interact with real‐time visual, auditory, or haptic representations of their brain activity using a brain–computer interface (BCI). Typically, the BCI targets specific neural indices (e.g., alpha power) with the goal to help improve a specific cognitive function (e.g., attention) [28, 29, 30]. Neurofeedback is commonly used in combination with electroencephalography (EEG) due to its high temporal resolution [31, 32]. For example, EEG‐based neurofeedback has been used with the aim to help improve cognitive performance [28, 33], sleep quality, and emotional control [34]. Neurofeedback can also be extended to a group of individuals (i.e., interbrain neurofeedback, or cross‐brain neurofeedback), providing feedback targeting indicators of multiple brains to enable dyads or group members to regulate interbrain coupling [35, 36, 37, 38, 39, 40, 41, 42, 43]. In most multibrain biofeedback studies to date, participants were explicitly instructed to try out strategies to maximize their interbrain coupling (“what does it mean to be on the same brain wavelength?”) and received real‐time feedback using a BCI designed to reinforce moments of increased interbrain coupling, often visualized through light patterns and avatar sizes/distances. These studies suggest that multibrain neurofeedback training may indeed lead to an increase in interbrain coupling, implying the potential for active induction of interbrain coupling through external training [36, 38]. While these findings are promising, they also leave some questions unaddressed. Most notably, it is unclear under which conditions interbrain neurofeedback can help improve social connectedness. For example, the observed increases in interbrain coupling may arise from the awareness that the BCI reflects their interbrain coupling. Alternatively, participants may actually be able to extract meaningful information from the BCI directly, effectively manipulating synchrony with their collective behavior. And if participants experience a sense of control, then what might be the associated internal mental processes that allow them to exert such control?

Here, we explicitly investigated the interplay between multibrain neurofeedback and social connectedness. Fifty‐seven dyads underwent a 12‐min interbrain neurofeedback, during which they imagined synchronizing their brain activity with each other. The BCI consisted of two line‐drawn brains approaching each other or separating from each other based on the level of synchronization [35]. Interbrain coupling was quantified in real time using online coherence of EEG signals. Critically, half of the dyads were presented with a BCI based on real neural signals and half were shown a BCI based on randomly generated signals (real‐neurofeedback [NFB] vs. sham‐NFB). Both groups received the same instruction that the BCI would reflect their interbrain coupling; this allowed us to test whether the realness of the data would bias their sense of joint control. Before and after the imagery task, participants completed subjective measurements evaluating their experience of social connectedness and shared mental processes.

We hypothesized that interbrain neurofeedback would enhance interbrain coupling (H1) and increase subjective feelings of social connectedness (H2), and that these effects would be mediated by shared mental processes—specifically shared intentionality and perceived similarity (H3). In addition, we included participants’ reported sense of joint control (of avatar distance) as a task‐specific measure of coagency during interbrain neurofeedback. While not conceptualized as a general pathway to social connectedness, joint control may serve as an experiential precursor that scaffolds the emergence of shared intentionality and/or perceived similarity. By integrating real‐time neural data, subjective reports, and mediation analyses, this study aims to advance understanding of how multibrain neurofeedback may enhance social connectedness through underlying cognitive mechanisms.

## 2 Methods

### 2.1 Participants

A total of 114 college students (36 males and 78 females, aged 20.08 ± 2.19 years, mean ± standard deviation) were recruited for the study. Dyads were then randomly assigned to either the real‐NFB (*n* = 28 dyads) or sham‐NFB group (*n* = 29 dyads) using a simple randomization procedure based on computer‐generated random numbers, without stratification. To mitigate the effects of gender and familiarity [44], each dyad consisted of two participants of the same gender who were previously unacquainted with each other. All participants were right‐handed, had normal or corrected‐to‐normal vision, and had no history of neurological or psychiatric disorders. Prior to the experiment, each participant provided informed consent and they were compensated with 60–70 yuan upon completion. This study was approved by the Ethical Institute Review Board of Shenzhen University.

### 2.2 Experimental Tasks and Procedures

**FIGURE 1:**
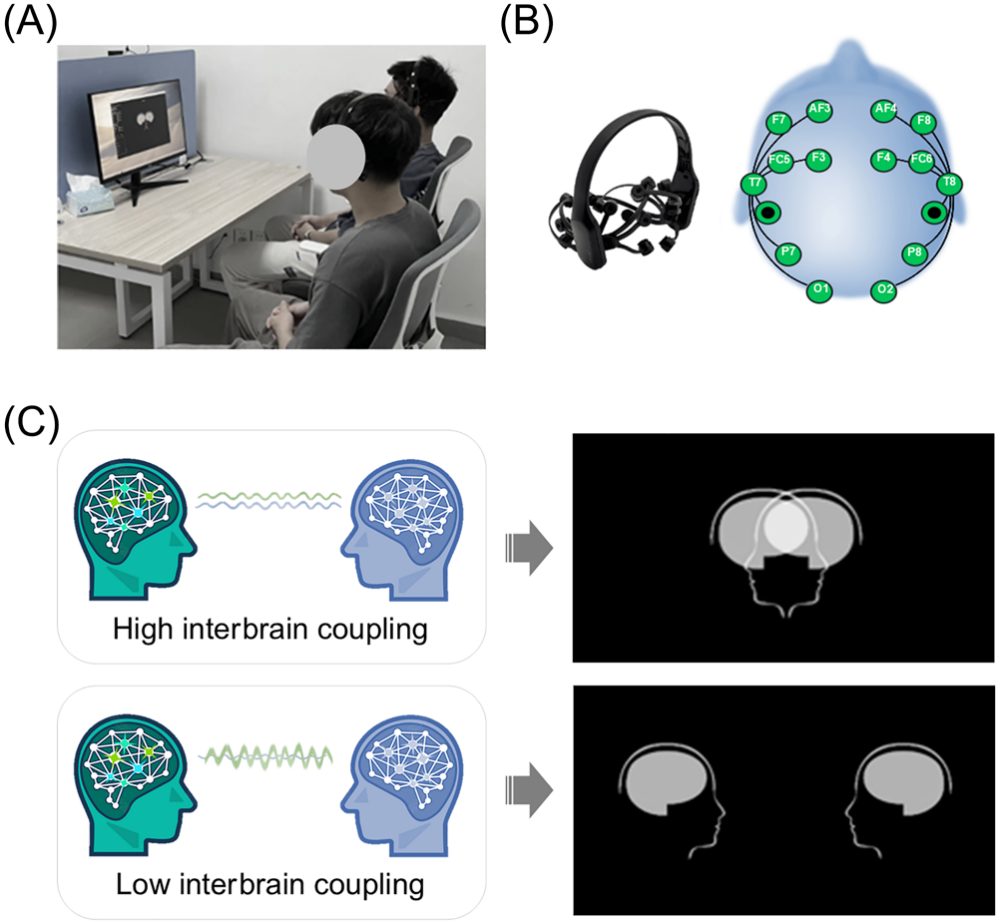
Interbrain neurofeedback setup. (A) Experimental setup. (B) Portable EEG device and electrodes configuration. (C) The overlap of the two avatar icons represents a higher interbrain coupling between the participants, while their separation indicates a lower interbrain coupling.

Two participants were seated next to each other (Figure 1A), in a quiet laboratory in front of a screen (DELL, 24‐inch, 1080p, 60 Hz). Each dyad was equipped with a portable wireless EEG headset (EMOTIV EPOC X; Figure 1B), for real‐time computation of interbrain coupling and visual feedback (Figure 1C).

**FIGURE 2:**
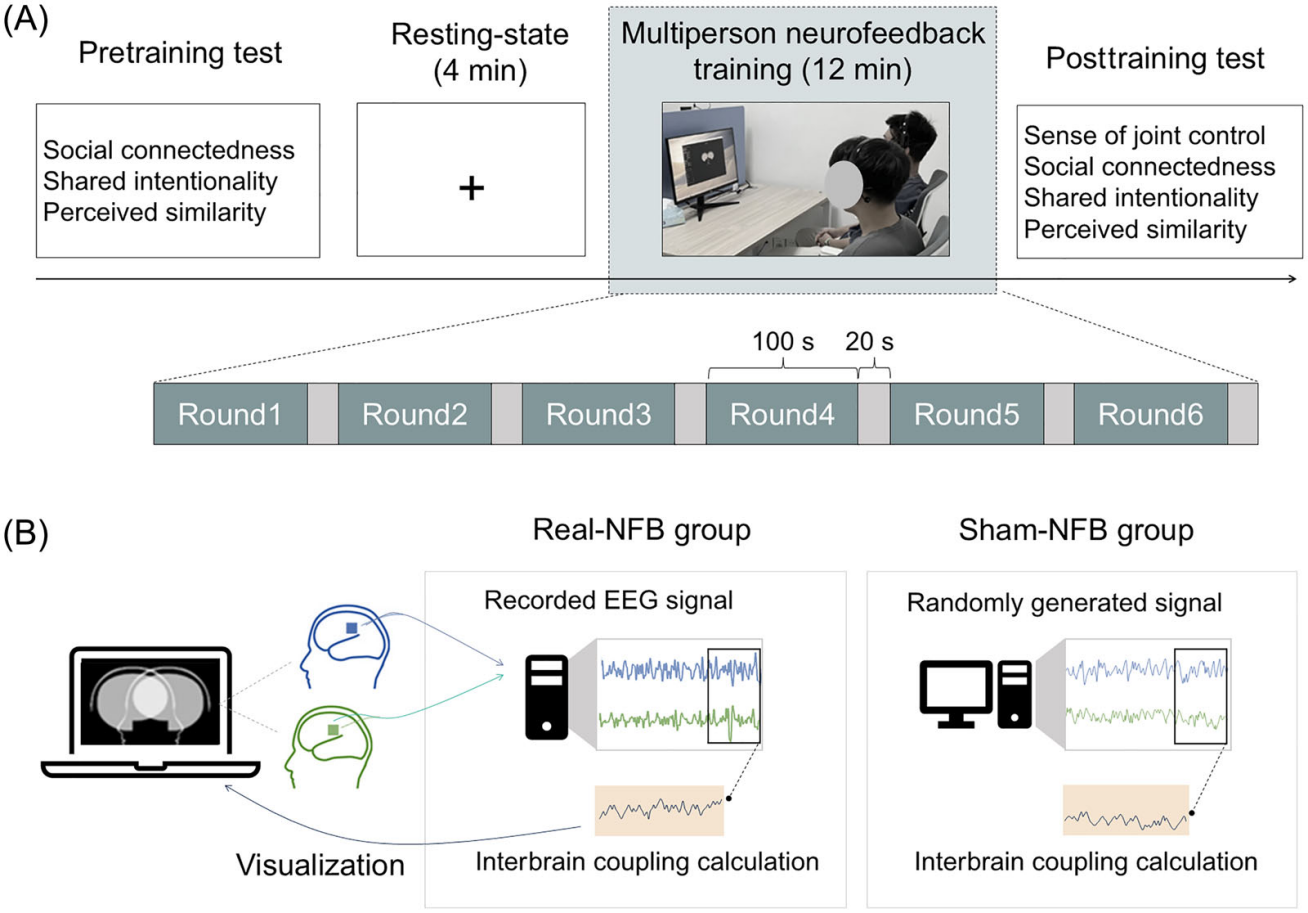
Experimental design. (A) Experimental procedure. Participants completed six rounds of interbrain neurofeedback training, each lasting 100 s, with a 20‐s break between rounds. (B) Group assignment and neurofeedback visualization. Participants were randomly assigned to the real‐neurofeedback (NFB) group or a sham control group. In the real‐NFB group, feedback was delivered according to the synchronization computed from the actual EEG signals of the participants. Conversely, in the sham‐NFB group, feedback was based on the synchronization calculated from data generated by the program.

During the experiment, dyads underwent four successive sessions: a pretraining test session, a 4‐min resting‐state session, a 12‐min interbrain neurofeedback training session, and a post‐training test session (Figure 2). The resting‐state session was deliberately scheduled after the pretraining test to reflect participants’ neural state immediately before neurofeedback. Although some cognitive processing related to questionnaire reflection may persist, this design minimizes confounds from questionnaire‐induced neural fluctuations during the task, enabling a cleaner baseline for neurofeedback effects. The details of each session are outlined below.

#### 2.2.1 Session I: Pretraining Test Session

Participants were asked to individually complete questionnaires measuring social connectedness and shared mental processes. During this session, no verbal or nonverbal communication was allowed.

##### 2.2.1.1 Social Connectedness

Based on previous studies [45, 46], four items from the emotional bonding questionnaire were extracted to evaluate participants’ feelings of social connectedness, including psychological closeness (i.e., “How close do you feel to your partner?”), friendliness (“To what extent do you feel you could be friends with your partner?”), likability (“How much do you like your partner?”), and trust toward their partner to their partner (“How much do you trust your partner?”) [46, 47]. All items were rated on a 9‐point Likert scale, ranging from 1 (not at all) to 9 (strongly). The results from the four items were averaged. Cronbach's alpha for the four‐item social connectedness scale at the pretraining test was 0.831, indicating good internal consistency.

##### 2.2.1.2 Shared Mental Processes

To evaluate participants’ shared mental processes, we used two sets of questionnaires to examine shared intentionality and perceived similarity during the task. Regarding shared intentionality, five items were drawn from the rapport questionnaire [20, 48]. Participants responded to statements such as: “During interaction with my partner, there was a shared flow of thoughts and feelings.” Cronbach's alpha for these items at pretraining was 0.876, indicating good reliability. For perceived similarity, five items were used [20, 49], with participants rating their degree of similarity to other participants. All questionnaire items were rated on a 9‐point Likert scale, ranging from 1 (not at all) to 9 (strongly). Cronbach's alpha for perceived similarity items at pretraining was 0.750, reflecting acceptable internal consistency. The results from the items for shared intentionality and perceived similarity were averaged.

#### 2.2.2 Session II: Resting‐State Session

Upon completing the pre‐experiment tests, participants engaged in a 4‐min resting‐state session. During this session, they were seated upright in front of a monitor and instructed to remain still and relaxed, without engaging in any particular thoughts. To capture neural dynamics under different attentional states, the session included two consecutive 2‐min conditions: eyes closed followed by eyes open [38]. In the eyes‐closed condition, participants were asked to close their eyes naturally and stay awake. In the eyes‐open condition, they were instructed to keep their eyes open and fixate on a centrally displayed cross. This design aligns with established EEG protocols [38, 50, 51], where eyes‐closed rest enhances intrinsic alpha activity by reducing visual input, and eyes‐open rest promotes alertness and minimizes drowsiness. Including both conditions improves the reliability and ecological validity of resting‐state EEG data, providing a robust baseline for assessing neurofeedback effects.

#### 2.2.3 Session III: Interbrain Neurofeedback Training Session

Participants underwent an interbrain neurofeedback training task. In this task, they viewed a BCI display featuring two avatar icons, with instructions that each represented their own brain. They were told that the proximity of the two icons on the screen varied based on the synchronization level of their brain activity; closer proximity indicated greater synchronization. In both groups, participants were asked to concentrate on pushing the icons closer together with their minds, aiming to synchronize their brain activity with their partner's. Example instructions (provided in Chinese) include “During the task, you will see two avatar icons on the screen representing your brains. Your goal is to push these avatars together with your minds so they overlap in the center of the screen. The icons will change in real time based on the synchronization between your brainwaves.” To achieve this, they needed to adjust their brainwaves to align with their partner's as closely as possible. This was an intentionally abstract instruction that was supposed to help the participants use different strategies. Consistent with recent practice [38], participants were not provided with any instructions regarding the different experimental conditions (real‐NFB vs. sham‐NFB), ensuring that they remained blind to the testing conditions. Only nonverbal interactions were allowed during this session, as verbal communication is more likely to introduce additional cognitive and emotional layers (e.g., language processing and emotional tone), which could complicate the interpretation of the neurofeedback. Each dyad completed six rounds of interbrain neurofeedback, each lasting 100 s, with a 20‐s break between rounds (Figure 2A). In the real‐NFB group, dyads were presented with a BCI based on real‐time neural signals; in the sham‐NFB group, dyads were shown a BCI based on the synchronization calculated from random data (i.e., random samples drawn from a uniform distribution between 0 and 1) generated by the Hybrid Harmony program [35] (Figure 2B).

#### 2.2.4 Session IV: Post‐Training Test Session

Following the interbrain neurofeedback training, participants were asked to complete post‐training surveys, which included questionnaires measuring social connectedness and shared mental processes (same as pretraining tests). At the post‐training test, Cronbach's alpha values indicated good internal consistency for social connectedness (0.876), shared intentionality (0.886), and perceived similarity (0.818). They also reported their sense of joint control during the task. Specifically, they responded to two questions regarding their perceived control over the movement of their avatar icon: “To what extent do you feel you can control the movement of your avatar icon?” and “To what extent do you feel you can control the movement of the avatar icon together?” Responses were rated on a 9‐point Likert‐type scale, ranging from 1 (did not feel in control at all) to 9 (felt totally in control). A mean score of the sense of control was computed based on those two questions. The measure of joint control is exploratory in nature. Although not drawn from a validated scale, they align conceptually with prior work on joint action and coagency [52].

### 2.3 EEG Recording and Online Neurofeedback Computation

During the task, the raw EEG signals of dyads were recorded using two 14‐electrode portable wireless EEG headsets (EMOTIV EPOC X), with a sampling rate of 128 Hz. Prior to recording, signal quality was verified using EMOTIV's built‐in EEG Quality Indicator. Electrodes were adjusted until all channels reached “green” status (≤10 kΩ), indicating stable contact and optimal signal acquisition according to manufacturer standards. The LabRecorder program was used to save data through Lab Streaming Layer (LSL; https://github.com/sccn/labstreaminglayer). LSL assigns high‐precision timestamps at the data source based on a shared system clock, ensuring submillisecond synchronization between data streams and minimizing the impact of wireless jitter.

Online interbrain coupling between individuals was computed in the Hybrid Harmony program [35] (Python 3.6), using a sliding 3‐s window. This approach allows for a smooth and responsive feedback signal, maintaining signal stability and providing sufficient data length for reliable coherence computation. Prior research has successfully used 3–4 s moving windows to capture meaningful fluctuations in social cognitive states during social interaction [38, 53]. The choice of a 3‐s window is also supported by our frequency bands of interest—estimating interbrain coupling at 1 Hz (within the delta band) requires capturing multiple cycles, typically over a span of 3–5 s.

The rate of the real‐time analysis was roughly 12 times per s in this study, depending on the computation bandwidth of the system running Hybrid Harmony. At each update cycle, the most recent 3 s of time‐aligned EEG data from the dyads were extracted and band‐pass filtered using an infinite impulse response (IIR) filter into canonical frequency bands [54]: delta (1–3 Hz), theta (4–8 Hz), alpha (9–12 Hz), and beta (13–30 Hz). The filtered signals were transformed via a Hilbert transform to compute the analytic signal. Coherence was calculated between homologous electrode pairs (e.g., Fp1–Fp1, C3–C3) across all frequency bands. These coherence values were averaged across the electrode pairs as well as the four frequency bands to produce a single coupling index for the current window, which was then used to drive the neurofeedback display [35]. Although no explicit online artifact correction (e.g., ICA) was applied due to real‐time constraints, temporal smoothing via the sliding window and multichannel averaging helped minimize the impact of transient noise and motion‐related fluctuations. This pipeline enabled real‐time feedback updates with smooth visual dynamics and sufficient signal stability.

The interbrain coupling values were then fed into a visualization algorithm [37]: higher interbrain coupling values brought the avatar icons closer together until they completely overlapped; conversely, lower interbrain coupling values resulted in the icons moving farther away (Figure 1C). Specifically, online interbrain coupling computation underwent a [0 0.3] min–max normalization [35] based on pilot data distributions, allowing even small fluctuations in coupling to result in visible avatar movement. The total latency from EEG acquisition to feedback display was 50–200 ms, with an initial 3‐s delay to initialize the analysis buffer and normalization baseline. This implementation ensured responsive and behaviorally meaningful feedback throughout the session. The overview and full protocol of implementing interbrain neurofeedback has been released publicly (https://github.com/RhythmsOfRelating/HybridHarmony).

### 2.4 Data Analysis

#### 2.4.1 Behavioral Data

For all behavioral data and subjective reports, the scores of participants in dyads were averaged to represent dyad‐level scores. Repeated‐measures ANOVAs were conducted on social connectedness and shared mental processes, with Group (real‐NFB vs. sham‐NFB) as the between‐dyad variable and Time (pretraining vs. post‐training) as the within‐dyad variable. When significant Group × Time interactions were observed, we conducted simple effects analyses with Bonferroni‐adjusted pairwise comparisons, implemented in SPSS. For sense of joint control, independent‐samples *t*‐tests were performed on post‐training data, with Group as the between‐dyad variable. Additionally, Pearson correlation analysis was conducted to investigate the relationship among social connectedness, shared mental processes, and sense of joint control. Specifically, we focused on the increase of social connectedness (Δ Social connectedness) and shared mental processes (Δ Shared intentionality, Δ Perceived similarity) from the pretraining to post‐training when performing correlation analysis. For all between‐group comparisons, Levene's test was first used to assess the equality of variances. When homogeneity of variances was violated, Welch's *t*‐test was applied, and the corresponding degrees of freedom were adjusted accordingly.

#### 2.4.2 EEG Data: Post Hoc Online Analysis

To assess the effectiveness of the neurofeedback manipulation, we conducted a post hoc analysis of the interbrain coherence values computed in real time during the training phase. In the real‐NFB group, feedback was based on participants’ actual EEG signals, whereas in the sham‐NFB group, feedback was driven by randomly generated signals. We therefore expected higher coherence values in the real‐NFB group. For each dyad, coherence values recorded during the neurofeedback phase were extracted and averaged across all training blocks. Group differences in mean coherence were then tested using an independent‐samples *t*‐test. This analysis was intended to verify that the neurofeedback manipulation produced distinguishable neural synchrony experiences across conditions.

#### 2.4.3 EEG Data: Offline Analysis

To parse true signals from both groups, we computed interbrain coupling using circular correlation coefficient (CCorr) analysis over the data post hoc (offline analysis). CCorr is commonly used to compute interbrain coupling in hyperscanning studies to capture phase relationships, in part because it is less sensitive to spurious correlations than some other metrics [55, 56, 57, 58]. CCorr is less suitable for real‐time analyses because of computational constraints that can induce temporal lags in the neurofeedback (i.e., reducing the “real‐time” nature of the experience). Offline analysis also provides the opportunity to preprocess the data more thoroughly, including the removal of artifacts and noise that might affect the calculation of interbrain coupling. This allows for the application of circular correlation to cleaner data, yielding more accurate and reliable results. To preprocess EEG data for the offline analysis, we used EEGLAB on MATLAB R2020a (MathWorks Inc., USA). Bad channels were excluded and an average re‐reference was applied to the raw EEG data [38]. A high‐pass filtering at 0.5 Hz was performed to eliminate slow fluctuations [36]. We performed independent component analysis (ICA) and then used the ADJUST toolbox implemented in EEGLAB to semiautomatically identify artifacts related to heart rate and eye blink [59, 60]. The effectiveness of ICA on EMOTIV devices has been previously validated [61]. For resting‐state analysis, 3‐s epochs were extracted for eyes‐open and eyes‐closed conditions using event markers. Epochs with motion artifacts or signal dropout were manually excluded through visual inspection.

Phase values were extracted from the preprocessed data using the Morlet wavelet transform on signals [11, 62] from 1 to 30 Hz, with a step size of 1 Hz. We calculated CCorr by using the Circular Statistics Toolbox to evaluate dyadic interbrain couplings [56, 58, 63]. CCorr measures the covariance between the phase time series of two neural signals. Specifically, it quantifies how consistently the phase of one signal changes in relation to the phase of another signal over time. Its function is defined as:

$$\mathrm{CCor}r_{\varphi ,\omega \;}=\frac{\Sigma_{k=1}^{N}\sin\left(\varphi_{k}-\bar{\varphi }\right)\sin\left(\omega_{k}-\bar{\omega }\right)}{\sqrt{\Sigma_{k=1}^{N}\sin^{2}\left(\varphi_{k}-\bar{\varphi }\right)\sin^{2}\left(\omega_{k}-\bar{\omega }\right)}}$$

where φ and ω represent the phase values of the two signals, while $\bar{\varphi }$ and $\bar{\omega }$ denote the mean phases of these signals. Following previous recommendations [63], the absolute CCorr was used as the indicator of interbrain coupling. This coefficient represents the absolute value of the Fisher *z*‐transformation of the circular correlation between two time series signals, that is, interbrain coupling = |*z*CCorr|.

Throughout the six rounds of the interbrain neurofeedback task, we discarded the last 10 s of each round to mitigate edge artifacts. The remaining data were used to compute task‐related interbrain coupling. First, we calculated the CCorr of every 3‐s interval across all possible electrode combinations (14 × 14) for each dyad and frequency [63]. This was done for both the neurofeedback session (Session III) and the resting‐state session (Session II). Then, interbrain coupling of each group was quantified by averaging CCorr values across all possible electrode combinations and time windows for each frequency [36]. Task‐related interbrain coupling was calculated by subtracting interbrain coupling at the resting‐state session from interbrain coupling at the interbrain neurofeedback session. In the remainder of the paper, we refer to task‐related interbrain coupling as interbrain coupling. These steps ultimately yielded 30 interbrain coupling values ranging from 1 to 30 Hz for each dyad.

Following interbrain coupling computation, we conducted 30 independent‐sample *t*‐tests, one for each 1‐Hz frequency bin from 1 to 30 Hz, to assess frequency‐specific differences in interbrain coupling between groups (real‐NFB vs. sham‐NFB). Multiple comparisons were correction using false discovery rate (FDR) at a threshold of *p* < 0.05. Additionally, Pearson correlation analysis was conducted to investigate the relationship between interbrain coupling and social connectedness, as well as shared mental processes. Specifically, we focused on the difference in subjective measurements (scores of social connectedness and shared mental processes) between the post‐training tests and the pretraining tests (“Δ Social connectedness”, “Δ Shared intentionality”, and “Δ Perceived similarity”).

## 3 Results

### 3.1 Validation of Interbrain Neurofeedback

**FIGURE 3:**
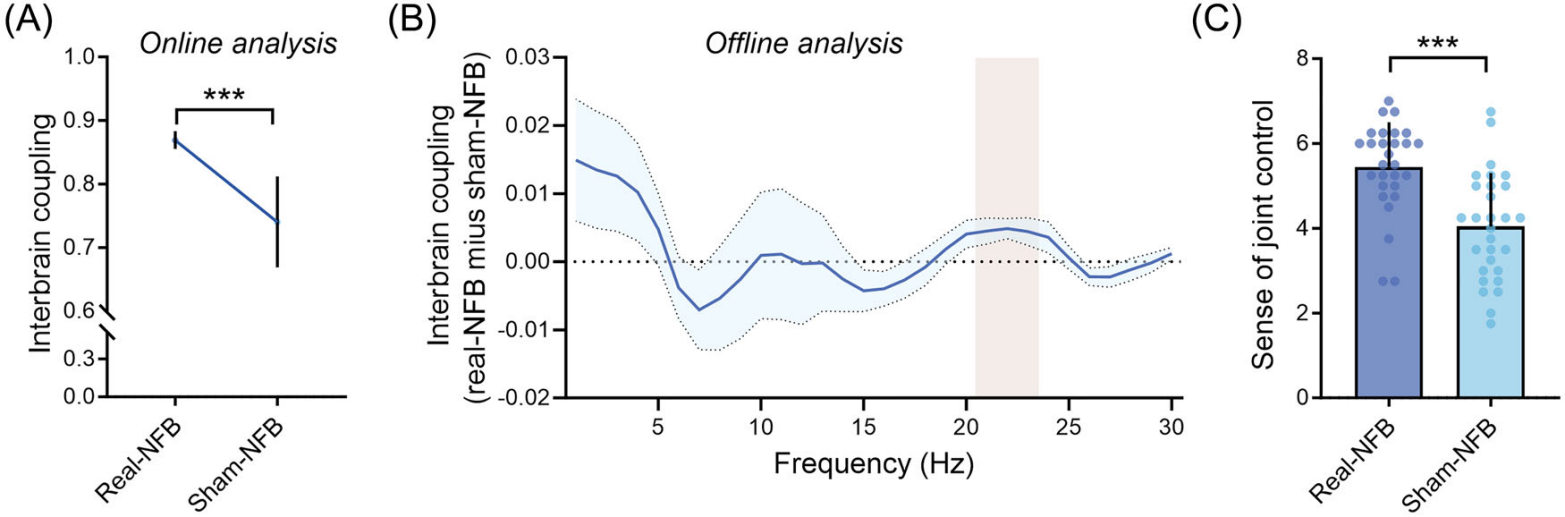
Validation of interbrain neurofeedback (NFB). The real‐NFB group elicited enhanced task‐related interbrain coupling in the online analysis (A), and in the offline analysis at 21–23 Hz, highlighted in a vertical bar (B). The shaded blue areas represent the standard deviation across dyads and are provided for descriptive purposes only. (C) The real‐NFB group demonstrated a higher sense of joint control compared to the sham‐NFB group. ^*^ *p* < 0.05, ^***^ *p* < 0.001. Error bars represent standard deviation.

As a first‐pass check, we examined whether interbrain neurofeedback training successfully induced interbrain coupling. As expected, the real‐NFB group demonstrated significantly higher interbrain coupling than the sham‐NFB group: Due to a significant result in Levene's test for equality of variances (*p* = 0.001), Welch's *t*‐test was used, *t* (25.65) = 4.979, *p* < 0.001, Cohen's *d* = 1.395 (Figure 3A). This indicates that the two groups experienced different proximities of the two icons on the screen (real‐NFB: relatively closer, sham‐NFB: relatively farther).

For the offline analysis, frequency‐wise independent‐sample *t*‐tests revealed that interbrain coupling in the real‐NFB group was significantly higher than that in the sham‐NFB group at 21–23 Hz (uncorrected *p*s < 0.05; Figure 3B). Interbrain coupling at 22 Hz survived after FDR correction (corrected *p* = 0.045). We then computed the averaged interbrain coupling for 21–23 Hz, and found that interbrain coupling at this frequency was higher in the real‐NFB group than that in the sham‐NFB group, *t* (55) = 2.931, *p* = 0.005, Cohen's *d* = 0.857. In the remainder of the manuscript, we focused on the offline interbrain coupling values.

Complementary to the above validation, we also assessed the training effects on the sense of joint control. Participants in the real‐NFB group reported a significantly higher sense of joint control compared to the sham‐NFB group (Figure 3C), *t* (55) = 4.542, *p* < 0.001, Cohen's *d* = 1.203. These results indicate that interbrain neurofeedback in the present study successfully induced stronger interbrain coupling, and participants in the two groups perceived different levels of the sense of joint control. These findings support our confirmatory hypothesis H1.

### 3.2 Effects of Interbrain Neurofeedback on Social Connectedness and Shared Mental Processes

**FIGURE 4:**
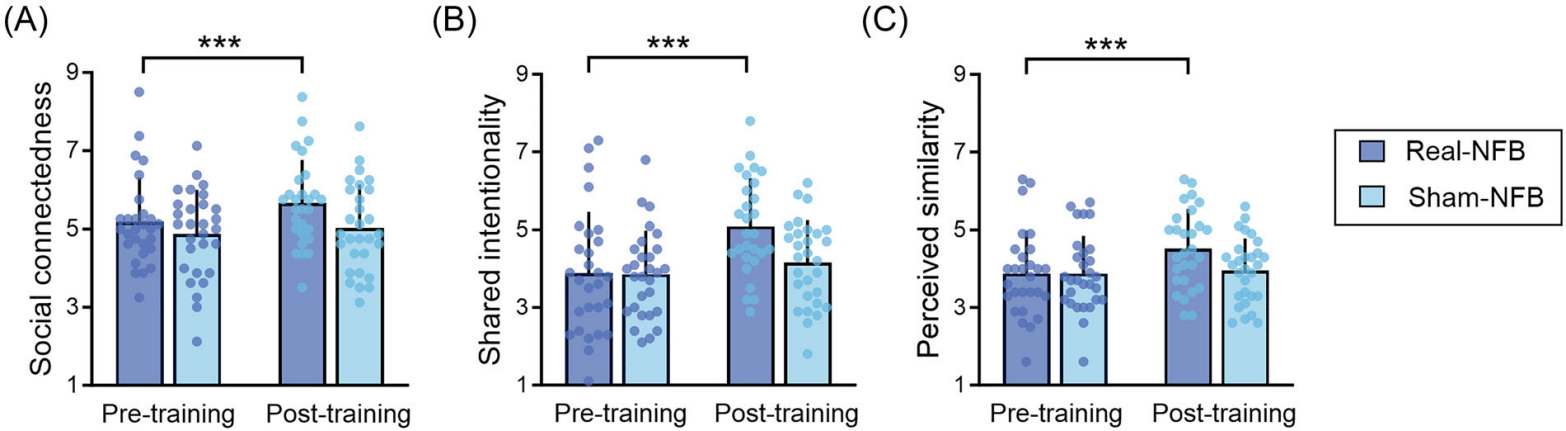
Effects of interbrain neurofeedback (NFB) on social connectedness and shared mental processes. The real‐NFB training promoted social connectedness (A), shared intentionality (B), and perceived similarity (C). ^***^ *p* < 0.001. Error bars denote standard deviations.

Having confirmed that interbrain neurofeedback successfully regulated interbrain coupling, we next tested our hypothesis H2, evaluating the effects of neurofeedback on social connectedness. The results showed that there was a significant main effect of Time (pretraining vs. post‐training), *F* (1, 55) = 18.992, *p* < 0.001, $\eta_{p}^{2}$ = 0.257, with enhanced social connectedness after neurofeedback. No main effect of Group (real‐NFB vs. sham‐NFB) was found, *F* (1, 55) = 2.646, *p* = 0.110, $\eta_{p}^{2}$ = 0.046. More importantly, we found a significant interaction effect, *F* (1, 55) = 5.695, *p* = 0.020, $\eta_{p}^{2}$ = 0.094, indicating that in the real‐NFB group, the post‐training session yielded a higher level of social connectedness compared with the pretraining session, *F* (1, 55) = 22.351, *p* < 0.001, $\eta_{p}^{2}$ = 0.289, while such effect was absent in the sham‐NFB group, *F* (1, 55) = 1.978, *p* = 0.165, $\eta_{p}^{2}$ = 0.035 (Figure 4A). These findings provide confirmation that interbrain neurofeedback can indeed predict an enhancement in social connectedness, thereby supporting our hypothesis H2.

To test our hypothesis H3, we first assessed whether interbrain neurofeedback could likewise promote shared mental processes (including shared intentionality and perceived similarity). For shared intentionality, the results revealed a significant main effect of Time, *F* (1, 55) = 29.253, *p* < 0.001, $\eta_{p}^{2}$ = 0.347, indicating a higher level of shared intentionality after neurofeedback training. No significant main effect of Group was detected, *F* (1, 55) = 2.438, *p* = 0.124, $\eta_{p}^{2}$ = 0.042. Notably, a significant interaction effect was observed, *F* (1, 55) = 10.598, *p* = 0.002, $\eta_{p}^{2}$ = 0.162. Specifically, in the real‐NFB group, shared intentionality significantly increased at post‐training compared to pretraining, *F* (1, 55) = 36.886, *p* < 0.001, $\eta_{p}^{2}$ = 0.401, while such observation was absent in the sham‐NFB group, *F* (1, 55) = 2.360, *p* = 0.130, $\eta_{p}^{2}$ = 0.041 (Figure 4B). Regarding perceived similarity, the main effect of Time was significant, *F* (1, 55) = 9.600, *p* = 0.003, $\eta_{p}^{2}$ = 0.149, showing that perceived similarity was enhanced after neurofeedback training. The main effect of Group was not significant, *F* (1, 55) = 1.487, *p* = 0.228, $\eta_{p}^{2}$ = 0.026. Moreover, there was a significant interaction effect on perceived similarity, *F* (1, 55) = 6.006, *p* = 0.017, $\eta_{p}^{2}$ = 0.098. In the real‐NFB group, perceived similarity significantly increased at post‐training compared to pretraining, *F* (1, 55) = 15.130, *p* < 0.001, $\eta_{p}^{2}$ = 0.216, while no such effect was observed in the sham‐NFB group, *F* (1, 55) = 0.213, *p* = 0.646, $\eta_{p}^{2}$ = 0.004 (Figure 4C). These findings suggest that interbrain neurofeedback also improved shared mental processes.

### 3.3 Interbrain Neurofeedback‐Regulated Interbrain Coupling Affects Social Connectedness Through the Sense of Joint Control and Shared Mental Processes

**FIGURE 5:**
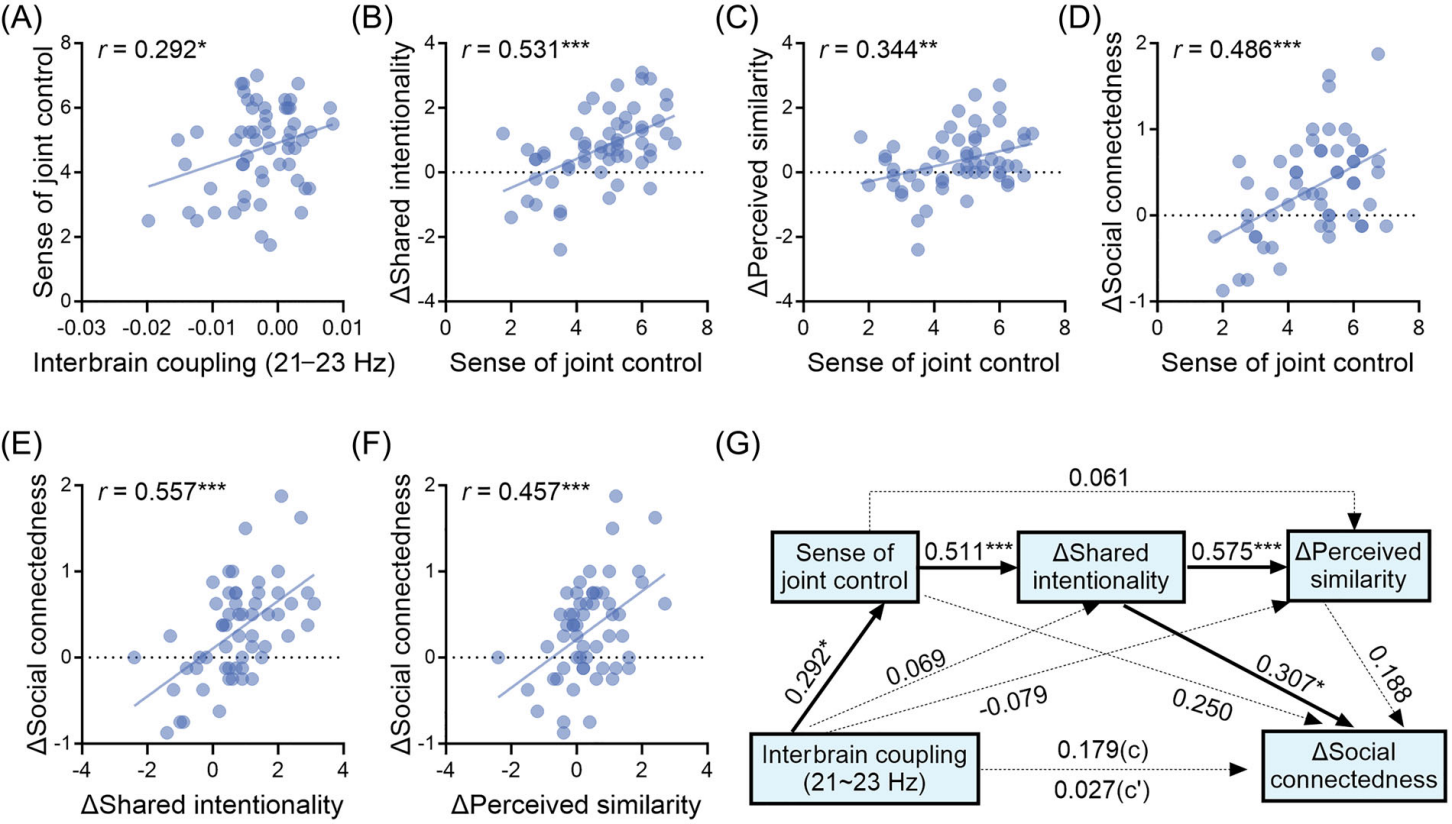
The relationship between interbrain coupling at 21–23 Hz, sense of joint control, shared mental processes, and social connectedness. (A) Interbrain coupling at 21–23 Hz was related to the sense of joint control. (B–D) Sense of joint control was associated with promoted shared intentionality (Δ Shared intentionality), promoted perceived similarity (Δ Perceived similarity), and social connectedness increase (Δ Social connectedness). (E, F) Promoted shared intentionality and perceived similarity predicted social connectedness increase. (G) The chain mediation result. Interbrain coupling at 21–23 Hz led to the increase of social connectedness through sense of joint control and the improvement of shared intentionality. Path coefficients were standardized. Δ = post‐training minus pretraining. ^*^ *p* < 0.05, ^**^ *p* < 0.01, ^***^ *p* < 0.001.

To explore the relationships among the interbrain neurofeedback‐regulated interbrain coupling, sense of joint control, shared mental processes, and social connectedness, we carried out a series of Pearson correlation analysis. We observed that interbrain coupling at 21–23 Hz was associated with a sense of joint control, *r* = 0.292, *p* = 0.028 (Figure 5A). Further, a sense of joint control was correlated with Δ Shared intentionality, *r* = 0.531, *p* = < 0.001 (Figure 5B), with Δ Perceived similarity, *r* = 0.344, *p* = 0.009 (Figure 5C), and with Δ Social connectedness, *r* = 0.486, *p* < 0.001 (Figure 5D). More importantly, both Δ Shared intentionality (*r* = 0.557, *p* < 0.001, Figure 5E) and Δ Perceived similarity (*r* = 0.457, *p* < 0.001, Figure 5F) were correlated with Δ Social connectedness.

Based on these pairwise associations, we conducted an exploratory chain mediation analysis, with interbrain coupling as the independent variable; sense of joint control, Δ Shared intentionality, and Δ Perceived similarity as potential mediators; and Δ Social connectedness as the dependent variable. This exploratory analysis revealed that interbrain coupling at 21–23 Hz affected the increase of social connectedness through the sense of joint control and the improvement of shared intentionality, standardized indirect effect = 0.046 (bootstrap sample = 5000), SE = 0.031, 95% CI = [0.0003, 0.1212] (Figure 5G). Bootstrap indirect effects of other paths were not significant (Table 1). To assess the robustness of this mediation structure, we also tested several alternative models—including parallel and reordered mediators (e.g., placing perceived similarity before shared intentionality). These analyses are reported in the Supporting Information. Importantly, across all model variants, the pathway from interbrain coupling to social connectedness via joint control and shared intentionality remained the most consistently supported.

**TABLE 1:** Standardized indirect effects of interbrain coupling on Δ Social connectedness.

Path	Effect	BootSE	95% CI
Interbrain coupling → Sense of joint control → Δ Social connectedness	0.073	0.089	[−0.036, 0.318]
Interbrain coupling → Δ Shared intentionality → Δ Social connectedness	0.021	0.038	[−0.010, 0.185]
Interbrain coupling → Δ Perceived similarity → Δ Social connectedness	−0.015	0.028	[−0.080, 0.033]
**Interbrain coupling → Sense of joint control →** **Δ Shared intentionality → Δ Social connectedness**	**0.046**	**0.031**	**[0.0003, 0.1212]**
Interbrain coupling → Sense of joint control → Δ Perceived similarity → Δ Social connectedness	0.003	0.009	[−0.010, 0.028]
Interbrain coupling → Δ Shared intentionality → Δ Perceived similarity → Δ Social connectedness	0.008	0.015	[−0.020, 0.042]
Interbrain coupling → Sense of control → Δ Shared intentionality → Δ Perceived similarity → Δ Social connectedness	0.016	0.014	[−0.007, 0.047]

*Note*: Indirect effects were estimated using bootstrapping with 5000 resamples, which provide robust confidence intervals and partially addresses multiple testing. However, no formal multiple comparison correction was applied across the multiple mediation pathways reported. Bold font indicates statistical significance.

Abbreviations: Δ, post‐training minus pretraining; CI, confidence interval; SE, standard error.

## 4 Discussion

The current study used an EEG‐based interbrain neurofeedback to investigate whether endogenously regulating interbrain coupling can promote social connectedness. We found that dyads who received interbrain neurofeedback training (compared to a sham group) showed an increase in interbrain coupling at 21–23 Hz. Task‐related interbrain coupling was correlated with dyads’ sense of social connectedness, while also improving the sense of joint control and shared mental processes (i.e., shared intentionality and perceived similarity), from pretraining to post‐training. Finally, a chain mediation analysis revealed that interbrain coupling at 21–23 Hz can predict increased social connectedness through the sense of joint control and the improvement of shared intentionality.

In contrast to previous interbrain neurofeedback studies [35, 36, 37, 38, 39, 40, 41, 42, 43], the present study introduces several methodological and conceptual advances. First, we implemented a rigorous randomized controlled design including a sham feedback group, which strengthens causal inference by controlling for nonspecific task engagement and expectancy effects. Second, we integrated multilevel psychological mediators—joint control, shared intentionality, and perceived similarity—to elucidate the cognitive and affective mechanisms linking interbrain coupling to social connectedness, an approach rarely addressed in previous work.

Our findings align with previous studies reporting a relationship between interbrain neurofeedback and social closeness and behavior [36, 37, 38]. It is important to note that although the training target range was broadly set between 1 and 30 Hz in the current study, offline analyses showed that the relevant frequency of interest was primarily concentrated at 21–23 Hz frequency band, within the beta frequency range. This may reflect a frequency‐specific effect. This high‐beta band (i.e., 21–23 Hz) might be more responsive to the neurofeedback protocol used, as the brain's intrinsic oscillatory properties may naturally favor this band under given training conditions. Previous studies have observed similar effects, suggesting that neurofeedback has frequency‐specific predictions for social behaviors [36]. This frequency‐specific coupling may reflect synchronized predictive processing between partners. Prior work has linked beta oscillations to motor simulation, top‐down attention, and predictive coding [64], as well as joint action and shared intentionality in social contexts [47, 65]. From this perspective, increased beta‐band interbrain coupling may signal alignment of internal forward models between individuals, enabling mutual prediction and coordination. This interpretation aligns with our mediation findings, in which joint control and shared intentionality linked neural coupling to increased social connectedness. Thus, beta‐band coupling may serve as a neurophysiological marker of dynamic cognitive alignment during interaction. It is not possible to completely rule out contributions of muscle artifacts to the beta coupling findings: the beta band is known to be susceptible to motor‐related signals, and while ICA‐based artifact rejection and across‐channel averaging were applied to mitigate artifact contamination, the lack of coverage over central sensorimotor areas precluded any topographical localization. We therefore caution against overinterpreting the anatomical origin of the beta effect. Future research using high‐density EEG or multimodal imaging could help isolate cortical contributions to interbrain coupling and rule out peripheral confounds.

Importantly, we observed that real interbrain neurofeedback enhanced social connectedness and shared mental processes (shared intentionality and perceived similarity). Further, our chain mediation analysis suggests that interbrain coupling regulated through interbrain neurofeedback may influence social connectedness through a sense of joint control and shared intentionality. This indirect pathway aligns with prior studies linking interbrain coupling to cognitive and affective states [66, 67, 68], as well as cooperative behavior and emotional resonance in dyads [69, 70, 71, 72, 73]. In our study, participants coordinated their thoughts to influence the avatar icons representing interbrain coupling, fostering a sense of joint control—a proximal psychological state reflecting coagency during the task. This shared sense of agency likely scaffolds the emergence of shared intentionality, reflecting mutual engagement toward joint goals [74]. Such cognitive and affective alignment promotes a feeling of connectedness, as individuals who experience coordinated intentions tend to feel more aligned and socially bonded [24, 75, 76]. Moreover, this alignment may further enhance social connectedness by reinforcing perceived similarity, which facilitates social support and affiliation [77, 78, 79, 80]. Taken together, our findings highlight how interbrain coupling serves as a foundational neurophysiological cue that scaffolds layered psychological processes—joint control and shared intentionality—that ultimately shape the subjective experience of social connectedness. This underscores the complex interplay between interbrain coupling, cognitive–motivational mechanisms, and social outcomes in the context of neurofeedback‐driven interpersonal interaction.

Given the novelty of our interbrain neurofeedback paradigm and the lack of prior effect size estimates for our sequential mediation model, we did not conduct an a priori power analysis. However, to assess the adequacy of our sample post hoc, we conducted a power analysis using G*Power based on the observed Group × Time interaction on social connectedness ($\eta_{p}^{2}$ = 0.094; *f* = 0.32). With a final sample of 57 dyads (114 participants), the estimated power was 0.796, which approaches the conventional 0.80 benchmark for adequate power. We acknowledge that our mediation model involved multiple sequential mediators—interbrain coupling, joint control, shared intentionality, and perceived similarity—introducing additional complexity. The observed standardized indirect effect (0.046, SE = 0.031) was small, and prior simulation studies suggest that larger samples are typically required to detect such effects with sufficient power [81]. Nonetheless, the indirect pathway remained statistically significant under bootstrap estimation (5000 samples), supporting its robustness. We emphasize that the mediation analysis was exploratory in nature. Future studies should include a priori power calculations based on the present effect sizes to guide confirmatory testing of these pathways.

The present findings also invite comparison with noninvasive brain stimulation methods such as transcranial electric stimulation, which have been shown to enhance interbrain coupling and modulate social behaviors [14, 15, 16, 82]. By directly altering cortical excitability, noninvasive brain stimulation can elicit changes in empathy and prosocial behavior [14, 82], and holds promise for both basic research and therapeutic applications [83, 84]. In contrast, neurofeedback involves endogenous regulation: participants actively adjust their neural activity using real‐time feedback, potentially offering a more naturalistic and self‐sustaining route to modulation. This may be particularly advantageous in social contexts where mutual responsiveness and perceived agency play central roles. Importantly, interbrain neurofeedback allows dyads or groups to coregulate shared neural states in real time, offering a direct way to train interbrain coupling itself. While conceptually distinct, neurofeedback and noninvasive brain stimulation may be viewed as complementary, providing different mechanisms, flexibilities, and intervention targets depending on the intended application.

In addition to its strengths, it is important to acknowledge several limitations in this study. First, the use of EEG‐based neurofeedback imposes restrictions due to its limited spatial resolution. Future investigations could explore the integration of EEG with functional near‐infrared spectroscopy (fNIRS), which offers better spatial resolution while keeping the naturalism. By combining these techniques, researchers can gain a more comprehensive understanding of both the temporal dynamics and spatial distribution of neural activity. Second, while interbrain neurofeedback showed promising group‐level results, this study solely relied on self‐reported measures of social connectedness. They may not fully capture the complexity and nuances of real‐life social relationships. Moreover, the symbolic visual feedback (i.e., converging brain icons) and the instruction to “imagine synchronizing your brains” may have contributed to participants’ subjective impressions of connectedness. While correlations between feedback coherence and connectedness were weak and nonsignificant in both groups (*r*s < 0.27, *p*s > 0.16), it remains possible that the symbolic feedback served as a reinforcing cue, particularly in the real‐NFB condition. Future work should explore the use of more abstract or affectively neutral feedback stimuli (e.g., avatar sizes), and incorporate post‐task assessments and expectancy‐matched controls to better isolate signal‐driven mechanisms from interpretive or placebo effects. Third, although the real and sham feedback streams were both rendered using the same visualization algorithm—ensuring smooth and visually plausible motion—the underlying neurophysiological relevance differed. While no participants expressed doubt about the feedback's authenticity during debriefing, future studies should incorporate more rigorous plausibility validation and expectancy control procedures to ensure perceptual equivalence and maintain engagement across conditions. Finally, although joint control and shared intentionality were included as mediators, these constructs were assessed concurrently with social connectedness at the post‐task time point. While framed to retrospectively capture experiences during neurofeedback and thus conceptually antecedent, the lack of temporal separation limits causal inference. Future research should dissociate mediator and outcome temporally, and incorporate behavioral or physiological indices to better capture social connectedness.

## 5 Conclusion

In summary, our study not only confirms the feasibility of endogenous regulation of interbrain coupling through interbrain neurofeedback but also adds to the existing body of literature by demonstrating that neurofeedback‐regulated interbrain coupling at 21–23 Hz can promote social connectedness, potentially through the sense of joint control and shared mental processes. Expanding on previous findings, our research underscores the important role that interbrain coupling plays in shaping social interactions and relationships. Moreover, our study highlights the potential of regulating interbrain coupling as a novel intervention strategy to optimize human social behaviors and foster healthier interpersonal connections, in both typical and psychiatric conditions [85, 86]. The short‐term modulation of interbrain coupling observed here may represent an early stage of interbrain plasticity [87, 88, 89, 90, 91]. Although the present study targeted immediate neurofeedback effects, longitudinal investigations are needed to assess whether repeated synchrony training produces durable enhancements in brain‐to‐brain coordination, which may underpin more stable social cognitive adaptations.

## Author Contributions

Conceptualization: Xiaojun Cheng, Suzanne Dikker, and Yafeng Pan. Methodology: Phoebe Chen and Suzanne Dikker. Data collection and analysis: Xiaojun Cheng, Rongbin Zhang, Ziyuan Song, and Feng Cheng. Writing – original draft: Xiaojun Cheng and Yafeng Pan. Writing – review and editing: Xiaojun Cheng, Suzanne Dikker, and Yafeng Pan.

## Conflicts of Interest

The authors declare no conflicts of interest.

## Supporting information




**Supporting Information**: nyas70135‐sup‐0001‐SuppMat.docx

## Data Availability

The data that support the findings of this study are available on request from the corresponding author.
