# Supplementary material for: Promoting Social Connectedness Through Interbrain Neurofeedback
Source: Ann N Y Acad Sci. 2025 Nov 10;1554(1):267–79. doi: 10.1111/nyas.70135 (PMC12728331; doi:10.1111/nyas.70135)
Supplement: Supplementary file 1 — Supporting Information: nyas70135‐sup‐0001‐SuppMat.docx [file NYAS-1554-267-s001.docx]

**Supplementary Materials**

**1. Items for shared intentionality**:

1) When I was interacting with my partner, there was a shared ﬂow of thoughts and feelings;

2) To what extent do you think you and your partner were aware of each other?

3) To what extent do you think you and your partner felt coordinated with each other?

4) To what extent do you think you and your partner understood each other?

5) To what extent do you think you and your partner had a feeling of mutual agreement?

**2. Items for perceived similarity**:

1) How much did you feel similar to your partner?

2) How much did you feel different to your partner?

3) Does your partner remind you of yourself in any way?

4) Do you think your partner is similar to you in character?

5) Do you think your partner likes the same musical styles that you do?

**3. Exploratory mediation models and results**

To further clarify the structure of psychological mechanisms linking inter-brain coupling to social connectedness, we conducted a series of exploratory mediation models using PROCESS (Hayes, 2013). These models tested alternative configurations and orders of the proposed mediators: joint control, shared intentionality, and perceived similarity.

**Model 1: Simple Mediation via Joint Control**

We first examined a simple mediation model in which joint control mediated the relationship between inter-brain coupling (21–23 Hz) and change in social connectedness. The indirect effect was statistically significant, standardized indirect effect = 0.138 (bootstrap sample = 5000), SE = 0.067, 95% CI = [0.013, 0.275], suggesting that the subjective experience of joint control may serve as a proximal mechanism by which inter-brain coupling shapes social connection (**Figure S1**).


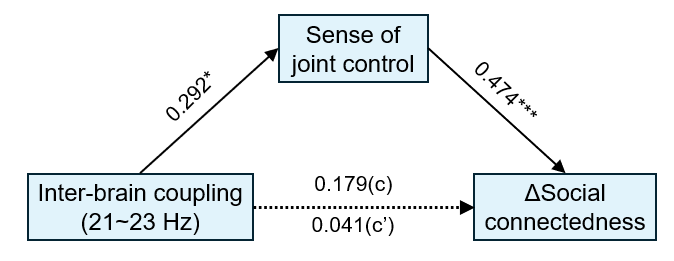


**Figure S1.** The mediation result. Path coefficients were standardized. Δ = post-training minus pre-training.  ^*^ *p* < 0.05, ^***^ *p* < 0.001.

**Model 2: Sequential Mediation via Joint Control and Shared Intentionality**

Next, we tested a three-path model including shared intentionality as a second mediator following joint control. Among all possible indirect paths, only the pathway “inter-brain coupling 🡪 joint control 🡪 shared intentionality 🡪 social connectedness” yielded a significant effect (**Figure S2**; **Table S1**). All other paths in the model were non-significant. This finding supports the idea that joint control facilitates the emergence of shared intentions, which in turn strengthen feelings of connection.


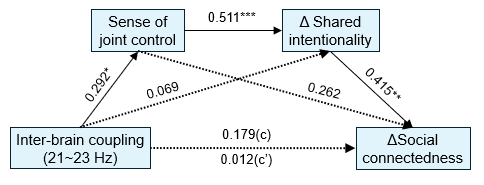


**Figure S2. The chain mediation (via joint control and shared intentionality) result.** Path coefficients were standardized. Δ = post-training minus pre-training. ^*^ *p* < 0.05, ^**^ *p* < 0.05, ^***^ *p* < 0.001.

**Table S1. Standardized indirect effects of inter-brain coupling on Δ Social connectedness via joint control and Δ shared intentionality.**

| Path | Effect | BootSE | 95%CI |
| --- | --- | --- | --- |
| Inter-brain coupling → Sense of joint control →  Δ Social connectedness | 0.076 | 0.050 | [-0.0034, 0.1838] |
| Inter-brain coupling → Δ Shared intentionality →  Δ Social connectedness | 0.029 | 0.047 | [-0.0648, 0.1243] |
| **Inter-brain coupling → Sense of joint control →**  **ΔShared intentionality → Δ Social connectedness** | **0.062** | **0.033** | **[0.0075, 0.1368]** |

*Note*. SE = standard error, CI = confidence interval. Δ = post-training minus pre-training. Bootstrap sample = 5000.

**Model 3: Sequential Mediation via Joint Control and Perceived Similarity**

We also tested a three-path model including perceived similarity as a second mediator following joint control. In this model, both “inter-brain coupling 🡪 joint control 🡪 social connectedness” and “inter-brain coupling 🡪 joint control 🡪 perceived similarity 🡪 social connectedness” paths were significant (**Figure S3**; **Table S2**). This suggests that perceived similarity may also mediate the relationship between inter-brain coupling and connectedness, though its effect may be broader and less process-specific than that of shared intentionality.


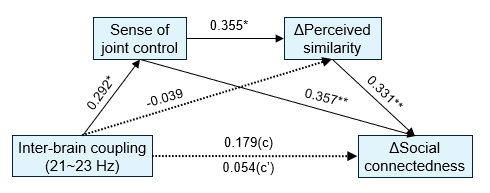


**Figure S3. The chain mediation (via joint control and perceived similarity) result.** Path coefficients were standardized. Δ = post-training minus pre-training. ^*^ *p* < 0.05, ^**^ *p* < 0.05, ^***^ *p* < 0.001.

**Table S2. Standardized indirect effects of inter-brain coupling on Δ Social connectedness via joint control and Δ perceived similarity.**

| Path | Effect | BootSE | 95%CI |
| --- | --- | --- | --- |
| **Inter-brain coupling → Sense of joint control →**  **Δ Social connectedness** | **0.104** | **0.057** | **[0.0046, 0.2284]** |
| Inter-brain coupling → Δ Perceived similarity →  Δ Social connectedness | -0.013 | 0.046 | [-0.1145, 0.0745] |
| **Inter-brain coupling → Sense of joint control →**  **Δ Perceived similarity → Δ Social connectedness** | **0.034** | **0.020** | **[0.0029, 0.0803]** |

*Note*. SE = standard error, CI = confidence interval. Δ = post-training minus pre-training. Bootstrap sample = 5000.

**Model 4: Sequential Mediation via Joint Control, Perceived Similarity and Shared Intentionality**

Finally, to examine the possibility that perceived similarity precedes shared goal alignment, we tested a four-path sequential model. In this model, only the path involving shared intentionality remained significant (i.e., “inter-brain coupling 🡪 joint control 🡪 shared intentionality 🡪 social connectedness”) (**Figure S4**; **Table S3**). This pattern further reinforces the central role of shared intentionality as a key link between inter-brain coupling and social connection, and suggests that while perceived similarity may be involved, it may not drive goal alignment in this context.


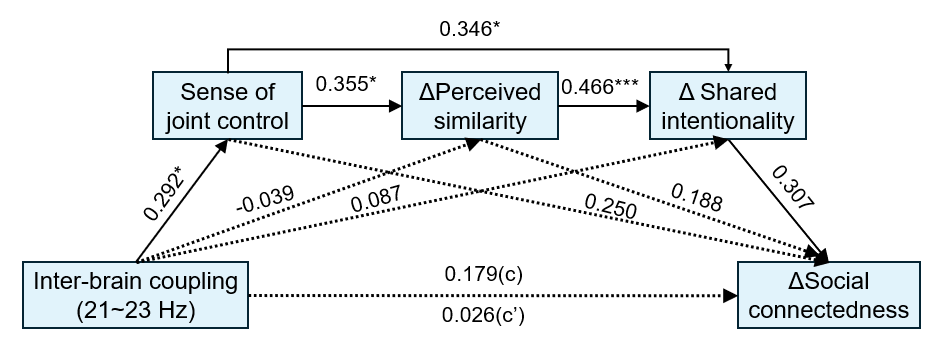


**Figure S4. The chain mediation (via joint control, perceived similarity and shared intentionality) result.** Path coefficients were standardized. Δ = post-training minus pre-training. ^*^ *p* < 0.05, ^**^ *p* < 0.05, ^***^ *p* < 0.001.

**Table S3. Standardized indirect effects of inter-brain coupling on Δ Social connectedness via joint control, Δ perceived similarity and Δ shared intentionality.**

| Path | Effect | BootSE | 95%CI |
| --- | --- | --- | --- |
| Inter-brain coupling → Sense of joint control →  Δ Social connectedness | 0.073 | 0.051 | [-0.0115, 0.1885] |
| Inter-brain coupling → Δ Perceived similarity →  Δ Social connectedness | -0.007 | 0.031 | [-0.0851, 0.0487] |
| Inter-brain coupling → Δ Shared intentionality →  Δ Social connectedness | 0.027 | 0.034 | [-0.0298, 0.1046] |
| Inter-brain coupling → Sense of joint control →  Δ Perceived similarity → Δ Social connectedness | 0.020 | 0.018 | [-0.0084, 0.0607] |
| **Inter-brain coupling → Sense of joint control →**  **Δ Shared intentionality → Δ Social connectedness** | **0.031** | **0.022** | **[0.0000, 0.0859]** |
| Inter-brain coupling → Δ Perceived similarity →  Δ Shared intentionality → Δ Social connectedness | -0.006 | 0.020 | [-0.0503, 0.0364] |
| Inter-brain coupling →Sense of joint control →  Δ Perceived similarity →Δ Shared intentionality →  Δ Social connectedness | 0.015 | 0.012 | [-0.0001, 0.0434] |

*Note*. SE = standard error, CI = confidence interval. Δ = post-training minus pre-training. Bootstrap sample = 5000.
